# An Incidental Finding of a Large Pheochromocytoma in an Asymptomatic Male: A Case Report

**DOI:** 10.7759/cureus.75659

**Published:** 2024-12-13

**Authors:** Joham Faryal, Jeyanthy Rajkanna, Samson O Oyibo

**Affiliations:** 1 Diabetes and Endocrinology, Peterborough City Hospital, Peterborough, GBR

**Keywords:** catecholamine hypersecretion, coronary artery thrombus, hypercoagulable state, myocardial infarction, pheochromocytomas

## Abstract

Pheochromocytoma is a rare neuroendocrine tumor that secretes excess catecholamines. Patients present with a classical triad of headache, palpitations, and sweating. If untreated, pheochromocytoma can result in life-threatening cardiovascular complications. Diagnosing pheochromocytoma is often challenging due to its atypical presentation, infrequent occurrence, and the fact that a small number of patients are asymptomatic, and very few pheochromocytomas are malignant. Here, we report a case of an 80-year-old man who was referred to the endocrine clinic after an incidental finding of an adrenal tumour. He did not have any symptoms related to pheochromocytoma. He had a 20-year complex cardiac history, including nine cardiac ischemic events, seven coronary artery stent placements, and a coronary bypass grafting procedure. He remained symptomatic, and coronary angiographic studies continued to demonstrate obstructed coronary arteries despite being on adequate secondary prevention. He also had type 1 diabetes, hypertension, and subclinical hypothyroidism. Both urinary and plasma catecholamines were elevated, confirming a diagnosis of pheochromocytoma. A computed tomography scan demonstrated a right subpleural mass and a left adrenal mass. A dedicated magnetic resonance imaging scan revealed a large left heterogenous adrenal lesion. A fluorodeoxyglucose positron emission tomography scan suggested that the subpleural mass and the adrenal mass could be malignant. He was commenced on medical treatment (alpha-blockade) and had no further ischaemic-related symptoms or cardiac events. Serial scans thereafter have indicated no increase in the size of both lesions. This case serves as a reminder and highlights the importance of asymptomatic pheochromocytoma.

## Introduction

Pheochromocytoma is an uncommon endocrine tumor originating in the adrenal glands, specifically in the chromograffin cells in the adrenal medulla. There is excessive secretion of catecholamines. The prevalence of pheochromocytoma is estimated to range between 1 in 4500 and 1 in 1700, with an annual incidence ranging from three to eight cases per one million people worldwide [1]. Nearly 5% of patients with incidentally discovered adrenal masses on anatomical imaging prove to have a pheochromocytoma [2]. The classical presentation includes paroxysmal hypertension, headaches, palpitations, sweating, and metabolic disorders [3]. Patients may also present with severe cardiovascular complications, such as ventricular tachycardia, myocardial infarction, heart failure, stress cardiomyopathy, and hypertensive crisis secondary to catecholamine-induced vasospasm or increased myocardial oxygen demand [4]. Measurement of plasma and urinary metanephrines, along with computed tomography scan or magnetic resonance imaging scan, is required for diagnosis and for planning treatment [2]. Adrenal tumors can also secret excess cortisol and aldosterone, which can lead to Cushing syndrome and Conn syndrome, respectively. These conditions should also be ruled out [5].

About 10% of patients with pheochromocytoma are asymptomatic or mildly symptomatic and discovered during an investigation of hypertension in the young [2,6]. Additionally, 10% of catecholamine-secreting pheochromocytomas are malignant and biochemically and histologically indistinguishable from benign tumors, the only difference being the presence of local invasion and distant metastases [6]. Other radiological features in favor of malignancy are tumor size (tumor size as an argument for malignancy is respectively by 20%, 65% and 89% for diameters ≥4 cm, ≥6 cm, and ≥8 cm), irregular contours, venous or contiguous invasion, presence of lymphadenopathy, a non-contrast density greater than 20 Hounsfield units (HU), delayed 15-minute contrast washout, and the presence of metastasis [6]. To characterize the lesion and presence of metastasis, a fluorodeoxyglucose positron emission tomography (FDG-PET) scan may be required [2]. Hence, it is important to have a full diagnostic work-up and management plan.

We report a man who was discovered to have a large, biochemically active pheochromocytoma while being investigated for an incidental subpleural lung mass during admission with pneumonia. He was asymptomatic despite elevated catecholamines.

## Case presentation

Medical history and demographics

An 80-year-old Caucasian man was referred to the endocrine outpatient clinic for investigation of an incidental adrenal adenoma, which was detected while being investigated for a subpleural mass found on contrast computerized tomography of the chest during admission with pneumonia. He denied ever experiencing symptoms such as headache, palpitations, sweating, or flushing. His medical history consisted of 20 years of symptomatic coronary artery disease, including nine myocardial ischemic events, seven coronary stent placements, and a coronary bypass grafting procedure. Previous coronary angiography demonstrated obstructed coronary arteries despite the absence of severe atherosclerotic disease. Thrombotic occlusions were suspected. He continued to have typical angina symptoms, alleviated by rest or nitro-glycerine spray. Further medical history included type 1 diabetes, hypertension, a previous toe amputation, chronic mild normocytic anemia, and intermittent compensated hypothyroidism. His medication regimen included insulin glargine, insulin aspart, losartan 50 mg daily, bisoprolol 2.5 mg daily, isosorbide mononitrate 120 mg daily, aspirin 75 mg daily, atorvastatin 20 mg daily, omeprazole 20 mg daily, and prasugrel 10 mg daily. He was a retired mechanical engineer, had been a lifelong non-smoker, and consumed less than one unit of alcohol per week. He had no family history of adrenal or any endocrine tumors.

On examination, he weighed 94.8 kg, with a height of 1.71 meters, and a body mass index of 32.4 kg/m^2^. His blood pressure was 145/70 mmHg (usual range: 140-150 mmHg systolic and 60-70 mmHg diastolic). A midline sternotomy scar from a previous coronary artery bypass operation was noted. No palpable swellings were detected in the neck and no cutaneous lesions. Visual field assessment and the rest of the systemic examination were unremarkable.

Investigations

Routine blood tests demonstrated normal renal function, liver function, and calcium levels. A thyroid function test demonstrated mild compensated hypothyroidism and a full blood count demonstrated mild normocytic anemia. A slightly elevated glycated hemoglobin level indicated inadequately controlled diabetes (Table 1).

**Table 1:** Initial blood test results.

Blood parameters	Results	Reference range
Sodium	135 mmol/L	133-146
Potassium	4.1 mmol/L	3.5-5.3
Chloride	107 mmol/L	95-108
Creatinine	95 µmol/L	59-104
Urea	7.4 mmol/L	2.5-7.8
Total bilirubin	12 µmol/L	<21
Alkaline phosphatase	97 U/L	30-130
Alanine transferase	16 U/L	10-60
Adjusted calcium	2.4 mmol/L	2.2-2.6
Thyroid stimulating hormone	4.52 mU/L	0.3-4.2
Free thyroxine	13.3 pmol/L	12-22
Glycated hemoglobin	62 mmol/mol	<48
Total cholesterol	4.3 mmol/L	<5
Low density lipoprotein cholesterol	2.1 mmol/L	<4
High density lipoprotein cholesterol	1.3 mmol/L	>1
Triglyceride	1.9 mmol/L	<2
Hemoglobin	120 g/L	130-180
White cell count	8.8 x 10^9^/L	4-11
Platelet count	293 x 10^9^/L	150-400

A 24-hour urinary metanephrines assessment revealed markedly elevated levels (metadrenaline and normetadrenaline), which suggested that the adrenal tumor was a pheochromocytoma. A normal 24-hour urinary-free cortisol level ruled out excessive cortisol secretion from the adrenal tumor (Table 2). Plasma renin and aldosterone levels were not assessed.

**Table 2:** 24-hour urinary metanephrines and free cortisol values. Indicating significantly raised urinary metanephrines with normal cortisol production.

Urinary parameters	Results	Reference range
24-hour urine volume for metanephrines assay	2299 ml	<3500
Urine creatinine	5428 µmol/L	3540-24600
24-hour metadrenaline excretion	6.4 µmol/24 hr	0.0-1.9
24-hour normetadrenaline excretion	6.5 µmol/24 hr	0.0-4.5
24-hour urine volume for cortisol assay	1501 ml	<3500
24-hour urine cortisol excretion	84 nmol/24 hr	<146

As the patient took a beta-blocker that could elevate urinary metanephrine levels, we also assessed plasma metanephrines (metadrenaline and noradrenaline), confirming excess production from a pheochromocytoma (Table 3). 

**Table 3:** Plasma metanephrines. Indicating significantly raised plasma metanephrines.

Plasma parameters	Results	Reference range
Plasma metadrenaline	1077 pmol/L	<600
Plasma normetadrenaline	3106 pmol/L	<1000

**Figure 1:**
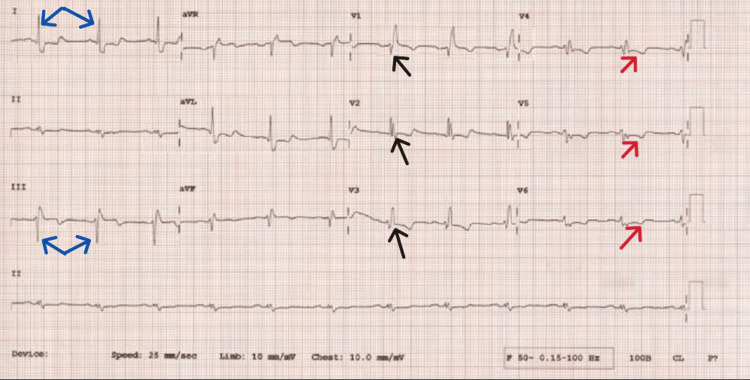
Electrocardiograph. Demonstrating partial right bundle branch block (black arrows pointing to RSR-patterns), left axis deviation (blue arrows in leads I & III) and generalised t-wave inversion (red arrows) in keeping with chronic ischemic changes.

An electrocardiogram indicated chronic ischaemic changes in the anterior and lateral leads, right bundle branch block with left axis deviation (Figure 1). An echocardiogram demonstrated mild dilatation of the left ventricle with eccentric hypertrophy. Systolic function was impaired with an ejection fraction of 40-45%, along with elevated filling pressures. Results were consistent with chronic ischemic heart disease and hypertension.

**Figure 2:**
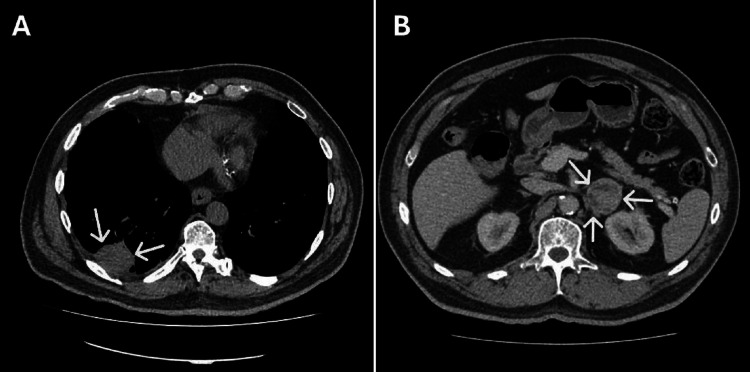
Radiological images of the chest and abdomen. A: High resolution computed tomography of the chest (transverse view) demonstrating a mass lesion measuring 7.0 cm x 6.1 cm (white arrows). B: Computed tomography scan of the adrenal glands (transverse view) demonstrating a large left inhomogeneous adrenal tumor measuring 4.3 cm x 4.9 cm (white arrows).

His initial computed tomography scan demonstrated a pleural-based mass-like lesion measuring 7.0 cm x 6.1 cm along the right diaphragmatic as well as the right costal pleura and an inhomogeneous left adrenal mass measuring 4.3 cm x 3.9 cm (Figure 2). Further scrutiny revealed that the adrenal mass was present (similar in size) on a computed tomography scan performed two years prior.

**Figure 3:**
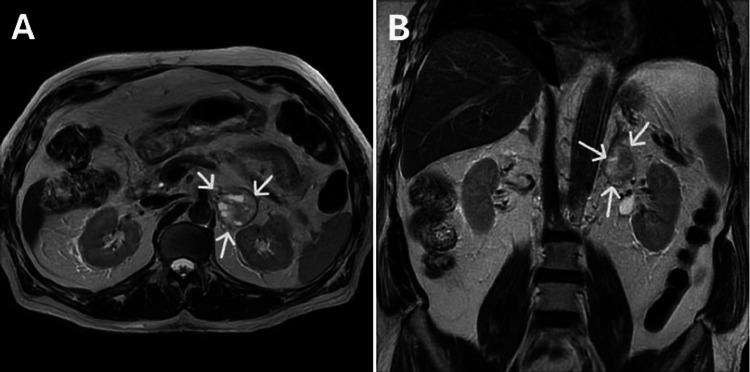
Magnetic resonance imaging scan of the abdomen. A: Magnetic resonance imaging scan of the adrenal glands (transverse view) demonstrating a lipid-poor left adrenal tumor, measuring 4.3 cm x 3.9 cm, and heterogenous in signal intensity (white arrows). B: Magnetic resonance imaging scan of the adrenal glands (coronal view) demonstrating the left adrenal tumor.

Based on the initial incidental finding of a large left adrenal lesion on the computerized tomography scan, a subsequent dedicated adrenal magnetic resonance imaging (MRI) scan was performed. The MRI confirmed a large left lipid-poor, heterogeneous adrenal lesion measuring 4.3 cm x 3.9 cm in size. There was no evidence of local invasion, lymphadenopathy, or distant metastasis (Figure 3). The combination of biochemical and radiological findings was consistent with pheochromocytoma. 

**Figure 4:**
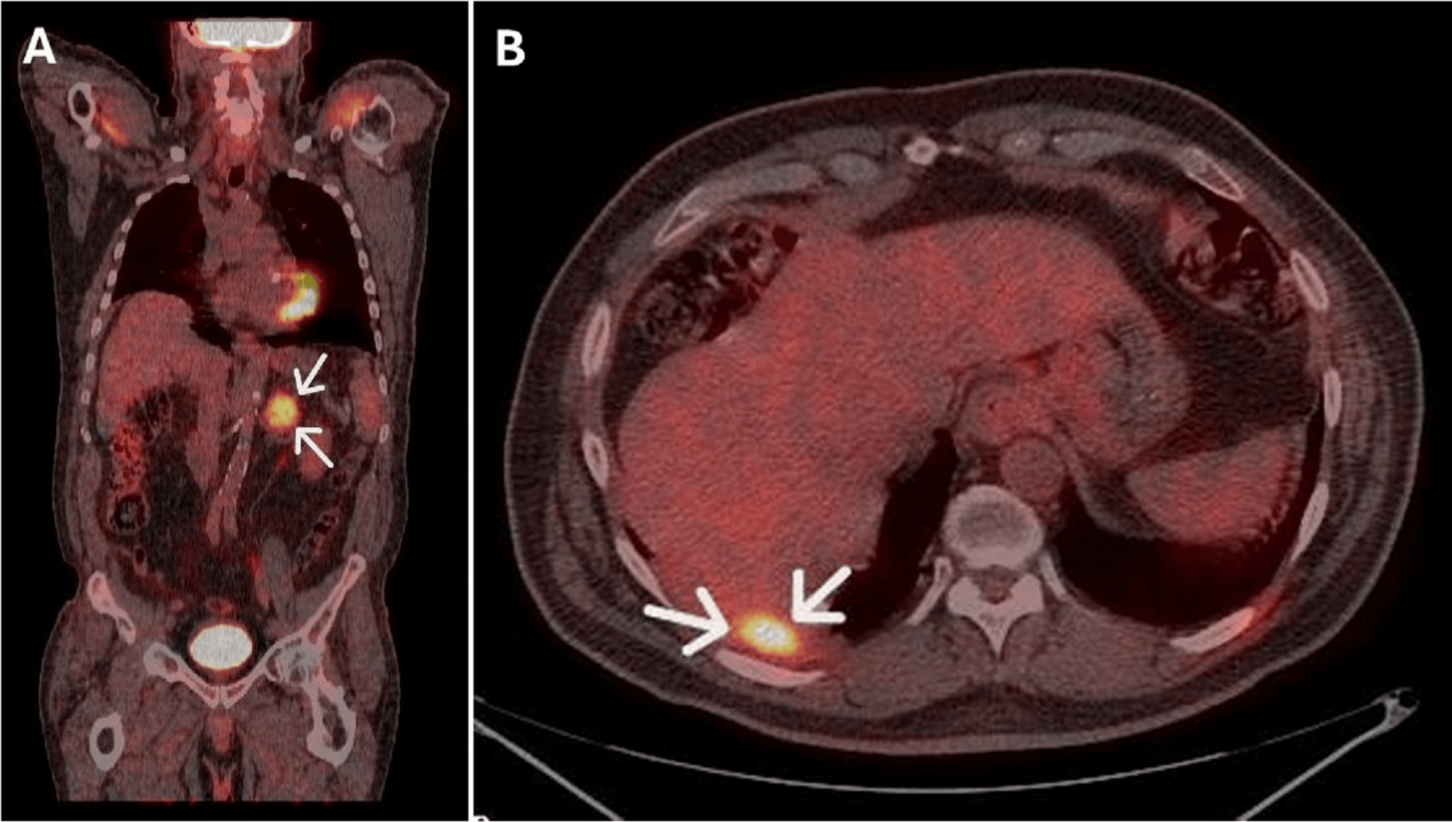
The fluorodeoxyglucose positron emission tomography (FDG-PET) scan of body from neck to thigh. A: Demonstrating high tracer uptake in the left adrenal gland lesion on coronal view (white arrows). B: Demonstrating high tracer uptake in the right subpleural mass and pleural thickening on transverse view (white arrows).

Despite having a computed tomography scan and a magnetic resonance imaging scan, the adrenal lesion was still classified as indeterminant. To characterize the lesion further, a fluorodeoxyglucose positron emission tomography (FDG-PET) scan was requested. There was increased tracer uptake in the left adrenal gland lesion and the right pleural lesion. There was no other abnormal uptake in the rest of the abdomen and no focal skeletal uptake. The FDG-avid uptake in the left adrenal lesion was suggestive of malignancy. This was the same for the right pleural lesion but was thought to be unrelated to the adrenal lesion (Figure 4).

Treatment

Because of the patient’s advanced age and multiple comorbidities, medical treatment as opposed to surgery was advised after a multidisciplinary team meeting. The tumor did not exhibit evidence of local invasion or distant metastasis. The patient was commenced on an alpha-adrenergic receptor blocker (doxazosin 2 mg daily) to be titrated according to blood pressure control. This continued alongside his usual secondary prevention treatment for ischemic heart disease. Additionally, the patient was referred to a cardiac rehabilitation program.

Outcome and follow-up

Following the optimization of medical treatment, the patient did not experience further cardiac events or chest pain. However, he did develop dizziness and postural hypotension when his doxazosin dose was titrated up to 6 mg daily. The patient felt better and had good blood pressure control on a dose of 2 mg daily.

He was to continue receiving regular follow-up care in the endocrinology and cardiology clinics, with annual imaging of the adrenal tumor and biochemical testing, including urinary cortisol and metanephrines. The lung multidisciplinary team concluded that the lung lesion was a sequelae of previous pneumonia and atelectasis rather than a tumor. Repeat computed tomography scans performed for two years have demonstrated a stable appearance, with no increase in the size of either the subpleural lesion or the adrenal mass.

## Discussion

Pheochromocytoma with life-threatening cardiovascular complications is both rare and atypical. Cardiac manifestations may include typical angina, electrocardiographic changes, arrhythmias, and myocardial infarction with elevated cardiac biomarkers. In some cases, transient systolic dysfunction of the left ventricle may occur, potentially resulting in heart failure and cardiogenic shock. Additionally, pheochromocytoma can present in the form of cardiomyopathy (Takotsubo or stress cardiomyopathy and catecholamine-induced cardiomyopathy). There have also been reports of pheochromocytoma manifesting as aortic dissection and peripheral ischemia [7-9]. The mechanisms underlying myocardial injury in this specific condition are mediated by the overly secreted catecholamines, which exert a direct toxic effect on cardiac myocytes and catecholamine-induced coronary vasoconstriction, resulting in a demand-supply imbalance and further cardiac muscle damage [7-9].

Multiple published reports have documented the occurrence of spontaneous thrombosis in association with pheochromocytoma, leading to the formation of systemic and intracardiac thrombi [10]. Despite this, angiographic evidence of occlusive thrombi in infarct-related coronary arteries of patients with pheochromocytoma is a rare finding. One such case reported findings of a high thrombus burden in the distal left anterior descending coronary artery secondary to a pheochromocytoma crisis. This patient required manual thrombus aspiration, with intracoronary injections of a thrombolytic and a vasodilatory agent [11]. The underlying mechanisms leading to spontaneous thrombosis in patients with pheochromocytoma are multifaceted and complex. Firstly, the excessive secretion of catecholamines can elevate levels of factor VIII and von Willebrand factor antigen and induce platelet activation and aggregation, thus promoting hypercoagulability [10]. Secondly, inflammatory cytokines and procoagulants produced by tumor cells, such as plasminogen activator inhibitor-1 (PAI-1), which is co-secreted with catecholamines, may contribute to hypercoagulability [12]. Thirdly, reduced coronary flow due to catecholamine-induced coronary vasospasm may also play a crucial role in inducing hypercoagulability [13].

The patient in this case report was of advanced age and had multiple risk factors for atherosclerotic heart disease (hypertension, diabetes, obesity, dyslipidemia). These risk factors could have co-contributed to ongoing angina symptoms [14]. In addition, the myocardial ischemic events had been going on for 20 years prior to the discovery of the pheochromocytoma. It is, therefore, unlikely that the pheochromocytoma was the cause of his ischemic heart disease, but the possibility of it being an exacerbation factor cannot be ruled out. Interestingly, the patient’s angina symptoms stopped occurring once commenced on alpha-blocker therapy.

Despite radiological evidence of the left adrenal tumor existing two years before, our patient did not exhibit the classic symptoms of a catecholamine-secreting pheochromocytoma (headache, palpitations, sweating, etc.). In addition, his blood pressure had been adequately controlled without any marked fluctuations or episodes of hypertensive crisis. As previously mentioned, about 10% of patients can be asymptomatic [2]. Our patient was already taking a beta-blocker for hypertension, and it is possible that this could have masked those expected symptoms. Asymptomatic pheochromocytoma also poses a dangerous situation when patients are going for any surgery involving the use of anesthetic drugs. Anesthetic drugs can exacerbate the life-threatening cardiovascular effects of catecholamines secreted by these tumors. A proportion of patients are diagnosed at the time of incidental surgery when induction of anesthesia has precipitated a hypertensive crisis, which is a high-mortality situation [15].

There are various treatment options available to manage the effects of catecholamine hypersecretion, including alpha-blockers, beta-blockers, calcium channel blockers, and tyrosine hydroxylase inhibitors, as well as surgical resection, radiofrequency ablation, and radiotherapy to the tumor [2]. Phenoxybenzamine, a long-acting, non-selective, non-competitive alpha-blocker, is commonly used for perioperative management. Other alpha-blockers such as doxazosin, terazosin, and prazosin may be utilized when phenoxybenzamine is unavailable or when the hypertension is not severe. Calcium channel blockers, including nifedipine, amlodipine, and verapamil, can also be used for additional blood pressure or symptom control, especially in some patients who cannot tolerate alpha-blocker side effects (e.g., headache, weakness, palpitations, sexual dysfunction, and postural hypotension). Beta-blockers such as propranolol, atenolol, or metoprolol are indicated in patients who are experiencing tachycardia, arrhythmias, or angina. However, beta-blockers should never be administered without effective α-blockade as their use in the absence of alpha-blockade can precipitate hypertensive crises and cardiac arrest [2]. Surprisingly, our patient was doing well on beta-blockers before the presentation.

Minimally invasive adrenalectomy (e.g., laparoscopic) is recommended for most adrenal pheochromocytomas. Open resection for large (e.g., >6 cm) or invasive pheochromocytomas is recommended to ensure complete tumor resection, prevent tumor rupture, and avoid local recurrence [2]. Patients with pheochromocytoma should be evaluated and treated by multidisciplinary teams at centers with appropriate expertise to ensure favorable outcomes [2]. Despite radiological pointers towards possible malignancy (large tumor >4 cm, heterogenous, avid FDG uptake, etc.), the scans did not indicate tumor invasion or distant metastasis. However, our patient was elderly, asymptomatic, and had multiple comorbidities that made surgery contraindicated. The multidisciplinary team recommended medical management with biochemistry and tumor size surveillance. This is a common option for patients who have non-malignant tumors, and surgery is contraindicated. As for patients with unresected malignant tumors, the 5-year survival rate is estimated at 40-77% [6]. The management of symptoms and blood pressure control is important in these patients. Our patient’s adrenal mass remained stable after two years of follow-up imaging, which would go against it being a malignant tumour.

New molecular targeted therapies (e.g., tyrosine kinase inhibitors, hypoxia-inducible factor 2 alpha [HIF2α] inhibitors, tumor vaccination, immune checkpoint inhibitors, antiangiogenic therapies, kinase signaling inhibitors) are being evaluated for treating patients with unresectable malignant and metastatic pheochromocytomas [16]. 

Genetic studies were not performed for our patient. Since 1990, 14 susceptibility genes have been reported. At least a third of patients with pheochromocytomas and paragangliomas have disease-causing germline mutation, and mutations in the succinate dehydrogenase complex sulfur subunit B (SDHB) gene lead to metastatic disease in at least 40% of affected individuals. Features that indicate a high likelihood of a hereditary cause are positive family history, syndromic features, and multifocal, bilateral, and metastatic disease. A clinical feature-driven diagnostic algorithm is recommended to establish the priorities for specific genetic testing [2].

## Conclusions

Pheochromocytoma is a rare neuroendocrine tumor that produces elevated plasma catecholamine levels, which, if untreated, can cause severe life-threatening cardiac complications. Asymptomatic pheochromocytoma is an unusual but serious condition, especially for patients undergoing an incidental surgical procedure in the presence of elevated catecholamine levels. Additionally, a small percentage of pheochromocytomas exhibit malignant behavior. Extensive diagnostic work-up in accordance with guidelines is required for an individualized management plan. Further research is required to raise awareness and identification of asymptomatic pheochromocytoma. This case serves as a reminder and highlights the dangers of asymptomatic pheochromocytoma.
